# Immune repertoire sequencing reveals differences in treatment response to camrelizumab plus platinum-based chemotherapy in advanced ESCC

**DOI:** 10.3389/fimmu.2025.1526443

**Published:** 2025-02-26

**Authors:** Xiaoling Zhang, Wenqi Zhao, Yunyi Du, Fei Su, Yuexiang Zhang, Hui Wang, Yongai Li, Min Liu, Yangjun Gao, Linlin Cai, Tingting Feng, Mei Wang, Chunmei Yao, Ning Xu, Yu Wang, Guohua Song, Wenqing Hu, Jun Zhao

**Affiliations:** ^1^ Department of Oncology, Changzhi People’s Hospital Affiliated to Changzhi Medical College, Changzhi, Shanxi, China; ^2^ Laboratory Animal Center, Shanxi Medical University, Taiyuan, Shanxi, China; ^3^ Department of Basic Medical Sciences, Shanxi Medical University, Taiyuan, Shanxi, China; ^4^ Statistics, Faculty of Science, University of Auckland, Auckland, New Zealand; ^5^ Department of Graduate School, Graduate of School of Shanxi Medical University, Taiyuan, Shanxi, China; ^6^ Imaging Center, Changzhi People’s Hospital Affiliated to Changzhi Medical College, Changzhi, Shanxi, China; ^7^ Department of Oncology, Anhui Huaibei Miners General Hospital, Anhui, China; ^8^ Department of Oncology, Fenyang Hospital of Shanxi Province, Fenyang, Shanxi, China; ^9^ Department of Pathology, Changzhi People’s Hospital Affiliated to Changzhi Medical College, Changzhi, Shanxi, China; ^10^ School and Hospital of Stomatology, Shanxi Medical University, Taiyuan, Shanxi, China; ^11^ Department of Gastroenterology, Changzhi People’s Hospital Affiliated to Changzhi Medical College, Changzhi, Shanxi, China

**Keywords:** camrelizumab, esophageal squamous cell carcinoma, immune repertoire sequencing, T-cell receptor beta-chain, immunoglobulin heavy chain, treatment response

## Abstract

This study evaluated the efficacy and safety of camrelizumab combined with platinum-based chemotherapy (taxanes [T] or fluorouracil agents [F] plus platinum [P] drugs) as the first-line treatment in advanced esophageal squamous cell carcinoma (ESCC), using immune repertoire sequencing (IRS) to explore treatment response mechanism. In this multi-center, prospective cohort study, 88 patients received camrelizumab plus TP or FP, achieving a 1-year progression-free survival of 56.8% and overall survival of 68.2%. The objective response rate (ORR) was 64.8%, with a disease control rate of 91.1%. While most treatment-related adverse events were mild, 12.5% of patients experienced grade ≥3 toxicities. IRS showed significant differences in T-cell receptor (TCR) β-chain and immunoglobulin heavy chain between patients with (ORR group) or without ORR (non-ORR group), particularly in the distribution and expression of some genes. Specifically, we found the significant differences in the amino acid composition of complementarity determining region 3 (CDR3) polypeptide sequences in TCR and B-cell receptor (BCR) between the ORR and non-ORR groups. For TCR, we observed substantial oligoclonal enrichment and differences in the abundance of specific V and J genes. Similarly, for BCR, we detected differences in the clonotype abundance of CDR3 polypeptide segments and identified several differential V genes. Camrelizumab combined with platinum-based chemotherapy is effective and well-tolerated as the first-line treatment for ESCC, and IRS may reveal mechanism influencing treatment response.

## Introduction

According to GLOBOCAN 2022, there were approximately 510,700 new cases of esophageal cancer (EC) and 445,100 deaths worldwide, making it the 11th most common cancer and the 7th leading cause of cancer-related deaths globally (1–3). Esophageal squamous cell carcinoma (ESCC) is the most common histological subtype of EC, accounting for over 85% of all cases (4).China is a high-incidence country for EC, with more than half of the patients being diagnosed at an advanced stage, leading to a poor prognosis (5–7).

The first-line treatment for advanced or metastatic ESCC primarily involves chemotherapy based on fluorouracil and platinum drugs. However, these treatments have limited efficacy, resulting in extremely poor prognosis with a 5-year survival rate of only 5% (8). Immune checkpoint inhibitors (ICIs) have been widely applied across various malignancies (9–12). Combining ICIs with chemotherapy has demonstrated improved outcomes for advanced ESCC (13–16). The findings from two landmark randomized controlled trials (RCTs), KEYNOTE-590 and ESCORT-1st, are particularly encouraging. The KEYNOTE-590 study revealed that the combination of pembrolizumab with 5-fluorouracil and cisplatin significantly prolongs overall survival (OS) in the first-line treatment of advanced EC, including 548 cases of ESCC (17). Camrelizumab, a highly selective and affinity-optimized PD-1 monoclonal antibody developed in China, was validated by the ESCORT-1st study. This study confirmed that camrelizumab combined with paclitaxel and cisplatin significantly extends progression-free survival (PFS) and OS in Chinese patients with advanced ESCC, offering a new approach for immunotherapy in EC (18).

In early 2020, we launched a real-world study (RWS) to evaluate the efficacy and safety of camrelizumab combined with platinum-based drugs as a first-line treatment for Chinese ESCC patients. This study is registered with Chictr.org.cn, (ChiCTR2000037942). The patient enrollment was nearly halted due to the significant impact of the COVID-19 pandemic in China that year. Despite the publication of the KEYNOTE-590 and ESCORT-1st study results a year after our study began, we decided to continue our research. This decision was made because we believe that RWS could provide essential supplementary insights to these two RCTs. Furthermore, we aimed to clarify the differences in clinical efficacy of camrelizumab combined with taxanes [T] or fluorouracil agents [F] plus platinum drugs [P], and sought to uncover the potential mechanisms behind these differences in a real clinical setting.

Immune repertoire sequencing (IRS) provides a direct reflection of lymphocyte-mediated immune responses (19, 20). In this study, we employed IRS to conduct an exploratory analysis of T-cell receptor (TCR) and B-cell receptor (BCR) diversity in patients with and without a significant treatment response. Through this analysis, we hope to provide a foundation for selecting the most appropriate treatment strategy for patients with ESCC.

## Methods

### Subjects and study design

This is a prospective, open-label, multi-center, multi-cohort study (ChiCTR2000037942), registered on September 4, 2020. The study was conducted in accordance with the Helsinki Declaration and international standards of good clinical practice. The Ethics Committee of Changzhi People’s Hospital approved the study protocol. All enrolled patients provided written informed consent.

In this study, we screened 96 patients with advanced HER-2 negative ESCC who received camrelizumab combined with chemotherapy from June 2020 to April 2023 across multiple centers. Eligible participants were newly diagnosed patients aged 18 years or older with histologically or cytologically confirmed ESCC. Inclusion criteria required patients to have at least one measurable target lesion per Response Evaluation Criteria in Solid Tumors (RECIST) v1.1 and an Eastern Cooperative Oncology Group (ECOG) performance status of 0-2. The location categories for esophageal cancer are classified according to the 8th edition of the AJCC/UICC Cancer Staging Manual (21). Exclusion criteria included the presence of secondary malignancies or other tumor types with brain or meningeal metastasis within three years prior to study initiation, as well as any concomitant diseases that, in the investigator’s judgment, could seriously endanger the patient’s safety or interfere with study completion.

Based on the patients’ responses to treatment, we further categorized them into two groups: the non-ORR group and the ORR group, which includes complete response (CR) and partial response (PR). All blood samples were collected prior to treatment. Peripheral blood T and B lymphocytes were isolated using lymphocyte separation fluid, and DNA was extracted. Ultimately, our study had 15 eligible blood samples from the non-ORR group for IRS. Therefore, we used simple random sampling to match 15 samples from the ORR group for IRS analysis. We captured the complementarity determining region 3 (CDR3) via multiplex PCR/5’RACE and performed high-throughput sequencing on the HiSeq 2000 platform. Sequencing background was filtered, and the resulting data were aligned to the IMGT immune receptor gene database to identify precise V (variable), D (diversity), and J (joining) gene segments and sequence loci. We conducted high-throughput sequencing of the CDR3 of T-cell receptor -chain (TRB)and immunoglobulin heavy chain (IGH) in 30 samples. The analysis of the IRS data included several aspects, such as statistical analysis of VDJ gene frequency, clone frequency distribution, and the number of peptide sequences.

First, we calculated the length distribution of CDR3 sequences to understand the proportion and distribution of sequences of varying lengths. Peptide sequences with the highest distribution percentages underwent amino acid analysis to characterize their roles in IR. The relative abundance and importance of different clonotypes in the immune response were assessed by calculating the abundance of CDR3 clonotypes. We analyzed the compositional diversity and abundance of the samples’ IRs using diversity metrics and statistical methods. Subsequently, we identified the V(D)J gene segments of each sequence by aligning the sequencing data with the TCR or BCR gene sequences in the reference database. We calculated the usage of different variable (V) and junction (J) segment genes to understand their frequency and preference within IR. Additionally, we observed specific connectivity patterns and significant connectivity events by examining the frequency of different V-J junctions.

### Treatment and follow-up

Camrelizumab combined with fluorouracil agents plus platinum (FP) or taxanes plus platinum (TP) is used as a first-line treatment regimen for ESCC. Patients undergo 4-6 cycles of chemotherapy, followed by maintenance therapy with camrelizumab alone.

Camrelizumab (200 mg) was administered intravenously on the first day of each 21-day cycle.

Platinum drugs used in the treatment included cisplatin, carboplatin, nedaplatin, or oxaliplatin. Cisplatin (25mg/m²) was administered intravenously to patients on days 1-3 of each 21-day cycle. Carboplatin (AUC=5) was administered on day 1 of each 21-day cycle. Nedaplatin (80mg/m²) and oxaliplatin (130mg/m²) were both administered intravenously on day 1 of each 21-day cycle.

Fluorouracil agents included 5-fluorouracil, S-1, and capecitabine. Patients received an intravenous bolus of 5-fluorouracil (400 mg/m²) on day 1, followed by intravenously continuous infusion at a dose of 2400 mg/²for 48 hours, every 21-day cycle. Capecitabine was administered orally at a dose of 1000 mg/m² twice daily (bid) on days 1-14. The dosage of S-1 was determined based on body surface area (BSA): for BSA between 1.25 and 1.5 m², patients took 50 mg bid orally on days 1-14; for BSA less than 1.25 m², the dose was 40 mg bid orally on days 1-14; and for BSA greater than 1.5 m², the dose was 60 mg bid orally on days 1-14.

The taxanes included paclitaxel, liposomal paclitaxel, and nab-paclitaxel (albumin-bound-paclitaxel). Paclitaxel and liposomal paclitaxel were administered intravenously at a dose of 135 mg/m², while nab-paclitaxel was administered at a dose of 260 mg/m², all on day 1 of each 21-day cycle.

The combination regimen continued until disease progression, intolerable toxicity, or completion of 4-6 treatment cycles. Maintenance treatment with camrelizumab was provided for up to two years to patients who completed 4-6 cycles of the combination therapy, as determined by the treating physician and the patient. No dose modifications of camrelizumab were permitted during this period. Patients could withdraw from the trial at any time without affecting their standard therapy or potential participation in other research studies. Investigators could discontinue a patient’s participation if further treatment was deemed harmful to their well-being. Efficacy evaluations were conducted every six weeks, including assessments of blood counts, liver and kidney function, tumor markers, and immune-related indicators. Enhanced CT scans of the chest and abdomen were also required. Safety profiles were evaluated at each visit. Detailed study protocols and the statistical analysis plan are provided in the **Supplementary Materials**.

### Outcomes

The primary outcome was progression-free survival (1-year PFS rate). Secondary outcomes included overall survival (1-year OS rate), defined as the time from the first dose of camrelizumab or chemotherapy to death from any cause, and objective response rate (ORR), which is the proportion of patients achieving a CR or PR as determined by computerized tomography. The disease control rate (DCR) was also assessed, defined as the proportion of patients achieving CR, PR, or stable disease (SD). For safety evaluation, adverse events (AEs) were reported according to the NCI Common Terminology Criteria for Adverse Events (CTCAE v5.0) throughout the study. Imaging and pathology assessments were confirmed by two clinical specialists. Responses were evaluated based on RECIST v1.1, and the toxicity profile included events occurring within 30 days after the end of therapy in all patients. As an exploratory objective, IRS results from 30 patients were analyzed.

### Statistical analysis

Statistical analysis was conducted using R studio (version 4.4.0; https://www.r-project.org/). A p-value of less than 0.05 indicated a statistical difference. Survival analysis was performed using Kaplan-Meier curves and the log-rank test. Subgroup analyses for within-group comparisons utilized the Fisher exact test. Safety outcomes were analyzed in patients who received at least one of the aforementioned doses of the study regimen. A multivariable Cox regression model was employed to assess the independent effect of subgroups on survival.

The length distribution of CDR3 polypeptides in the two groups was analyzed, and the differences in clonotype values of CDR3 polypeptide sequences of each length between groups were assessed using the Mann-Whitney U test. The t-test was used to compare the mean sequence length of the CDR3 region between the two groups. For peptide sequences in non-ORR group and ORR group, the corresponding CDR3 peptide sequences were extracted and analyzed for amino acid composition, which was then visualized using sequence logos created by WebLogo. Differences in the number of clonotypes between the two groups were also analyzed using the t-test. For the diversity indices—d50Index, Shannon norm, and Inverse Simpson—differences between the two groups were analyzed using the t-test, Mann-Whitney U test, and Mann-Whitney U test, respectively. Differences in the expression frequencies of all V/J genes between the two groups were examined using the Mann-Whitney U test.

## Results

### Characteristics of patients

**Figure 1:**
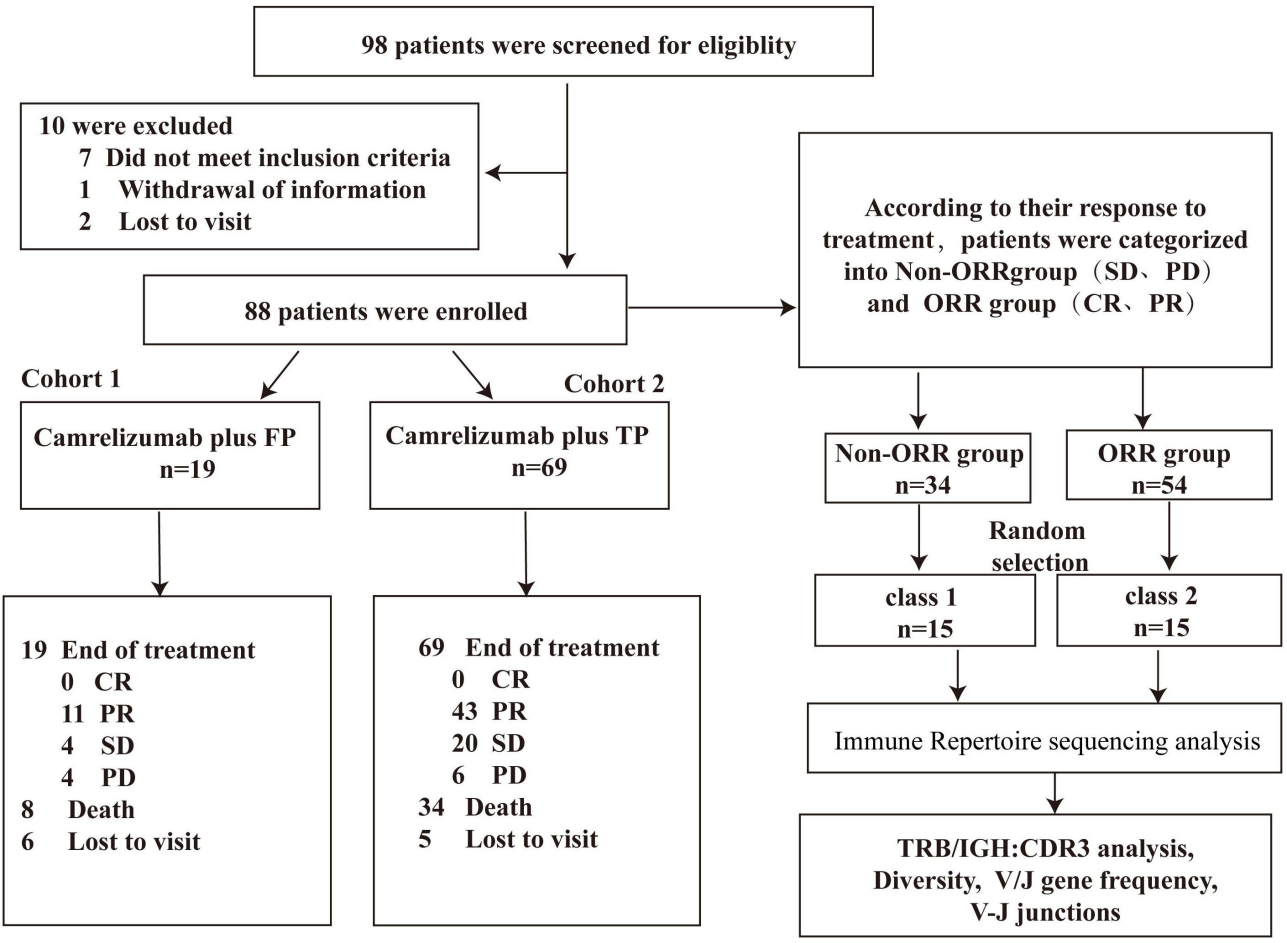
The flowchart of the study.

Between June 2020 and April 2023, we screened 98 patients diagnosed with locally advanced and advanced ESCC. A total of 88 patients were enrolled, as depicted in **Figure 1**. Among these, camrelizumab was administered in combination with FP to 19 patients, and with TP to 69 patients. As of the data cutoff on May 30, 2024, the median follow-up was 15.9 months (95% CI: 0.3-48.0) and the median OS was 23.2 months (95% CI: 16.4-not reached). Fifty-nine patients (67.0%) underwent 4-6 cycles of combined therapy, while the remaining 29 patients (33.0%) received fewer than 4 cycles. The median treatment duration was 4.1 months (range: 0.3-25.6) for chemotherapy and 5 months (range: 1-36) for camrelizumab. Overall, 35 patients (39.8%) continued follow-up without evidence of disease progression. The baseline characteristics of the patients are shown in **Table 1**.

**Table 1:** Patient baseline characteristics.

Characteristics	Total (N=88)	Camrelizumab plus TP (n= 69)	Camrelizumab plus FP (n=19)
Median age, years	69 (42-83)	69 (46-83)	69 (42-83)
< 65, n (%)	28 (31.8%)	23 (33.3%)	5 (26.3%)
≥ 65, n (%)	60 (68.1%)	46 (66.7%)	14 (73.7%)
Sex, n (%)			
Male	53 (60.2%)	41 (59.4%)	12 (63.2%)
Female	35 (39.8%)	28 (40.6%)	7 (36.8%)
ECOG			
0	18 (20.5%)	15 (21.7%)	3 (15.8%)
1	69 (78.4%)	54 (78.2%)	15 (84.2%)
2	1(1.1%)	0(0)	1(5.2%)
Location Category(33)			
Upper	20 (22.7%)	16 (23.2%)	4 (21.1%)
Middle	30 (34.1%)	18 (26.1%)	12 (63.2%)
Lower	38 (43.2%)	35 (50.7%)	3 (15.8%)
Pathological stage, n (%)			
Stage III	30 (34.1%)	24 (34.8%)	6 (31.6%)
Stage IV	58/88 (65.9%)	45 (65.2%)	13 (68.4%)
Histological grading, n (%)			
Low differentiated	4 (4.5%)	3 (4.3%)	1 (5.3%)
Moderately differentiated	70 (79.5%)	55 (79.7%)	15 (78.9%)
Highly differentiated	8 (9.1%)	6 (8.7%)	2 (10.5%)
Uncertainty	6 (6.8%)	5/69 (7.2%)	1 (5.2%)
Distant metastatic site, n (%)			
Lung	7/58 (12.1%)	5/45 (11.1%)	2/13 (15.4%)
Liver	14/58 (24.1%)	12/45 (26.7%)	2/13 (15.4%)
Lymph node	38/58 (65.5%)	29/45 (64.4%)	9/13 (69.2%)
Others	8/58 (13.8%)	7/45 (15.6%)	1/13 (7.7%)
PD-L1, n (%)			
< 1	17 (19.3%)	13 (18.8%)	4 (21.1%)
1-49	47 (53.4%)	39 (56.5%)	8 (42.1%)
≥ 50	4 (4.5%)	3 (4.3%)	1 (5.3%)
Uncertainty	20 (22.7%)	14 (20.3%)	6 (31.6%)
Duration of treatment, months	4.1 (0.3-25.6)	4.8 (0.3-25.6)	3.2 (13-24.2)
Immunotherapy cycle, n	5 (1-36)	5 (1-36)	4 (1-28)
Camrelizumab plus chemotherapy, n (%)			
4-6 cycles	59 (67.0%)	49 (71.0%)	10 (52.6%)
< 4 cycles	29 (33.0%)	20 (29.0%)	9 (47.4%)

ECOG, Eastern Cooperative Oncology Group; TP, platinum-based chemotherapy (taxanes plus platinum); FP, platinum-based chemotherapy (fluorouracil agents plus platinum).

Location Category (33) [the 8th edition of the AJCC/UICC cancer staging manuals].

### Efficacy

**Figure 2:**
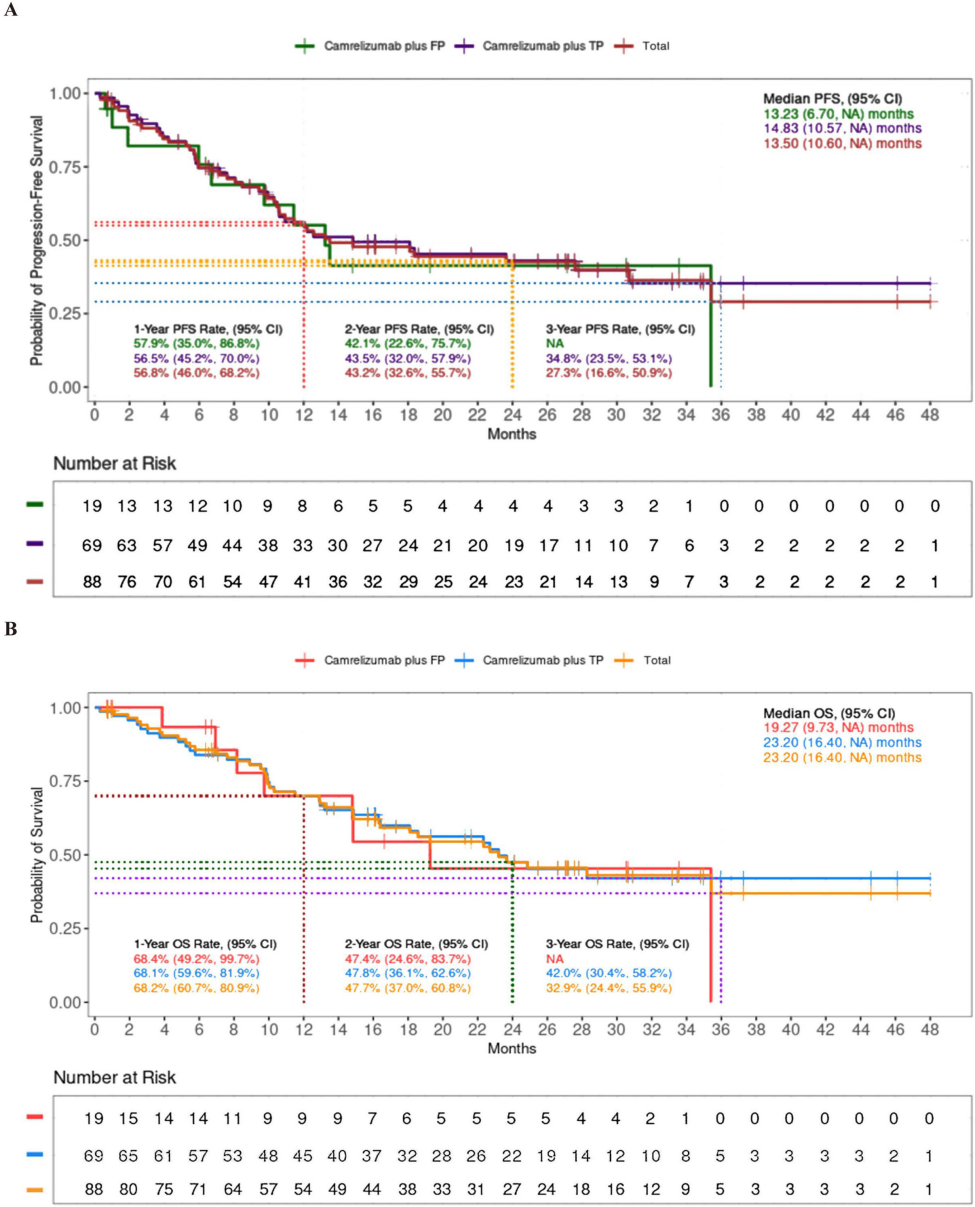
Progression-free survival (PFS) **(A)** and overall survival (OS) **(B)** in camrelizumab plus FP or TP.

The 1-year PFS rate was 56.8%, and the 1-year OS rate was 68.2%. The 2-year rate was 43.2% for PFS and 47.7% for OS. Notably, after 3-year follow-up, 33% of the patients were still alive (**Figure 2**, **Table 2**). No significant differences were observed between the TP and FP regimens in the 1-year PFS (56.5% vs 57.9%, p=1.000) and OS rates (68.1% vs 68.4%, p=1.000), or the 2-year PFS and OS rates (both p=1.000).

**Table 2:** Primary and second outcomes.

Outcomes	Total (N=88)	Camrelizumab plus TP (n=69)	Camrelizumab plus FP (n=19)	p
PFS rate				
1-year	50 (56.8%)	39 (56.5%)	11 (57.9%)	1.000
2-year	38 (43.2%)	30 (43.5%)	8 (42.1%)	1.000
3-year	24 (27.3%)	24 (34.8%)	NR	NR
OS rate				
1-year	60 (68.2%)	47 (68.1%)	13 (68.4%)	1.000
2-year	42 (47.7%)	33 (47.8%)	9 (47.4%)	1.000
3-year	29 (33.0%)	29 (42.0%)	NR	NR
Objective response rate (ORR)	57 (64.8%)	43 (62.3%)	11 (57.9%)	0.793
Disease control rate	81 (91.1%)	63 (91.3%)	159 (79.0%)	0.213
ORR group, n (%)	54 (61.4%)	43 (62.3%)	11 (57. 9%)	0.793
complete response	0 (0)	0 (0)	0 (0)	1.000
partial response	54 (61.4%)	43 (62.3%)	11 (57.9%)	0.793
Non-ORR group, n (%)	34 (38.6%)	26 (37.7%)	8 (42.1%)	1.000
stable disease	24 (27.3%)	20 (29.0%)	4 (21.1%)	0.573
progressive disease	10 (11.4%)	6 (8.7%)	4 (21.1%)	0.213

TP, platinum-based chemotherapy (taxanes plus platinum); FP, platinum-based chemotherapy (fluorouracil agents plus platinum); PFS, progression-free survival; OS, overall survival; NR, not reached.

**Figure 3:**
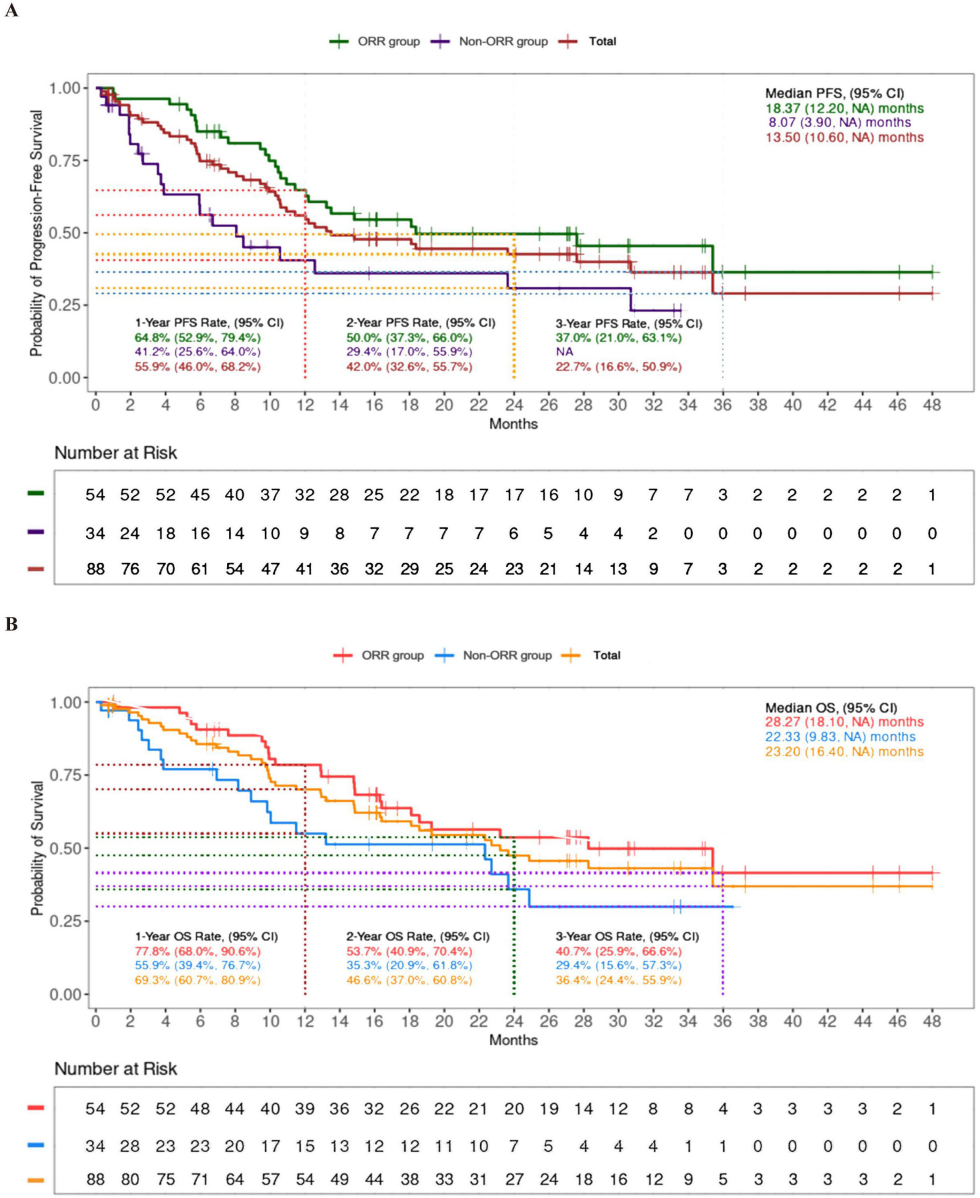
Progression-free survival (PFS) **(A)** and overall survival (OS) **(B)** between ORR group and non-ORR group.

The ORR was 64.8%, and the DCR was 91.1% (**Table 2**). For patients receiving the TP or FP regimen, there were no statistical differences in ORR (62.3% vs 57.9%, p=0.1779) and DCR (91.3% vs 79.0%, p=0.1005). Patients achieving ORR (the ORR group) had prolonged median (PFS of 18.37 months compared to 8.07 months in those who did not (the non-ORR group), as well as extended median OS of 28.27 months versus 22.33 months (**Figure 3**).

The subgroup analyses demonstrated that the number of chemotherapy cycles significantly influenced patients’ OS (**Supplementary Figure S1**) and PFS (**Supplementary Figure S2**). Specifically, patients receiving 4-6 cycles of chemotherapy had improved OS and PFS, while those receiving fewer than 4 cycles faced a higher risk of death and disease progression. Although other factors-such as age, sex, ECOG performance status, esophageal lesion location, and PD-L1 expression levels-did not demonstrate statistical differences, their trends were noteworthy. For instance, certain subgroups, such as older patients or specific sex, though not reaching statistical significance, displayed potential impacts on OS or PFS. These findings could inform hypotheses for further research, particularly with larger datasets.

### Safety

Treatment-related adverse events (TRAEs) were generally well-tolerated, with most being grade 1-2 in severity (**Table 3**). Grade 3 or 4 toxicities occurred in 12.5% (11/88) of patients, with no reported deaths. The most common AEs included reactive cutaneous capillary endothelial proliferation (RCCEP) (15.9%), fever (9.1%), and neutropenia (6.8%).

**Table 3:** Treatment-related adverse events.

	Total (N=88)	Camrelizumab plus TP (n= 69)	Camrelizumab plus FP (n=19)
Any AE, n (%)	57 (64.8%)	48 (69.6%)	9 (47.4%)
≥ Grade 3	11 (12.5%)	10 (14.5%)	1 (5.3%)
< Grade 3	46 (52.3%)	38 (55.1%)	8 (42.1%)
SAE	8 (9.1%)	8 (11.6%)	0 (0)
Treatment-related death, n (%)	1 (1.1%)	1 (1.4%)	0 (0)
Frequency of AE, n (%)				
RCCEP, n (%)	14 (15.9%)	11 (15.9%)	3 (15.8%)
Fever, n (%)	8 (9.1%)	8 (11.6%)	0 (0)
Neutropenia, n (%)	6 (6.8%)	6 (8.7%)	0 (0)
Thrombocytopenia, n (%)	4 (4.5%)	2 (2.9%)	2 (10.5%)
leukopenia, n (%)	4 (4.5%)	2 (2.9%)	2 (10.5%)
Anemia, n (%)	4 (4.5%)	2 (2.9%)	2 (10.5%)
Pneumonia, n (%)	4 (4.5%)	3 (4.3%)	1 (5.3%)
Nausea/vomiting, n (%)	4 (4.5%)	3 (4.3%)	1 (5.3%)
Rashes, n (%)	3 (3.4%)	3 (4.3%)	0 (0)
Diarrhea, n (%)	1 (1.1%)	1 (1.4%)	0 (0)
Hyperthyroidism, n (%)	1 (1.1%)	1 (1.4%)	0 (0)
Bipedal numbness, n (%)	1 (1.1%)	1 (1.4%)	0 (0)
Esophageal fistula, n (%)	1 (1.1%)	1 (1.4%)	0 (0)

AE, adverse event; SAE, serious adverse event; RCCEP, reactive cutaneous capillary endothelial proliferation.

### Immune repertoire

#### TCR repertoire analysis

**Figure 4:**
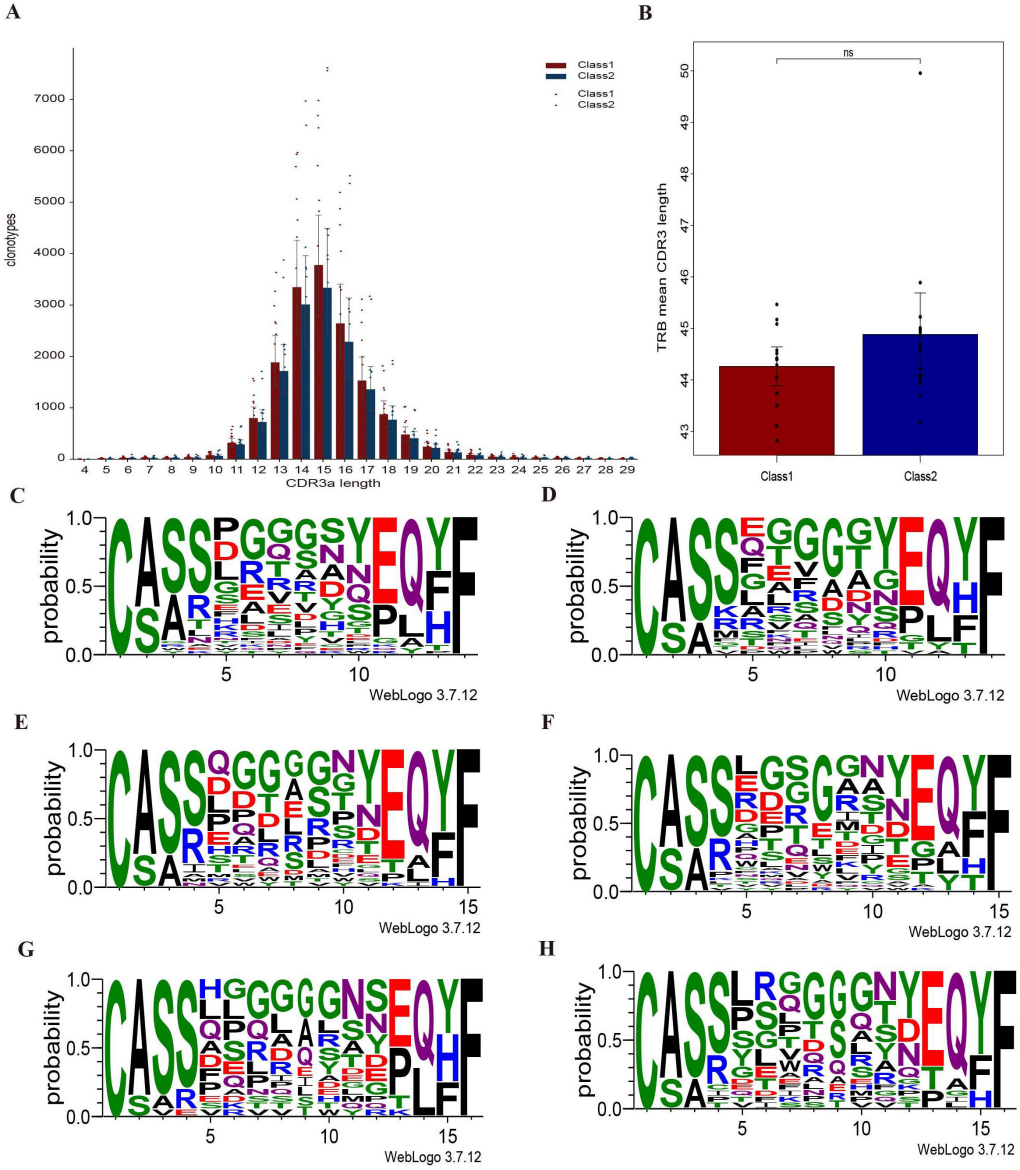
Comparison of TCRβ-CDR3 polypeptide sequences between non-ORR group (class 1) and ORR group (class 2). **(A)** Distribution of CDR3 peptide sequence length between groups. **(B)** Comparison of the length distribution of CDR3 peptide sequences between groups. **(C)** Amino acid sequence composition of CDR3 peptides of length 14 in non-ORR group. **(D)** Amino acid sequence composition of CDR3 peptides of length 14 in ORR group. **(E)** Amino acid sequence composition of CDR3 peptide of length 15 in non-ORR group. **(F)** Amino acid sequence composition of CDR3 peptide of length 15 in ORR group. **(G)** Amino acid sequence composition of CDR3 peptides of length 16 in non-ORR group. **(H)** Amino acid sequence composition of CDR3 peptides of length 16 in ORR group. NS, Not Significant.

We found differences in the amino acid composition of CDR3 polypeptide sequences between the ORR and the non-ORR groups within T-cell receptor β-chain (TRB). Notably, there was substantial oligoclonal enrichment (**Figure 4**). The most common TCR β-CDR3 sequences ranged from 14 to 16 amino acids in these patients. Additionally, the sequence pattern of the first four residues was quite similar between the two groups.

**Figure 5:**
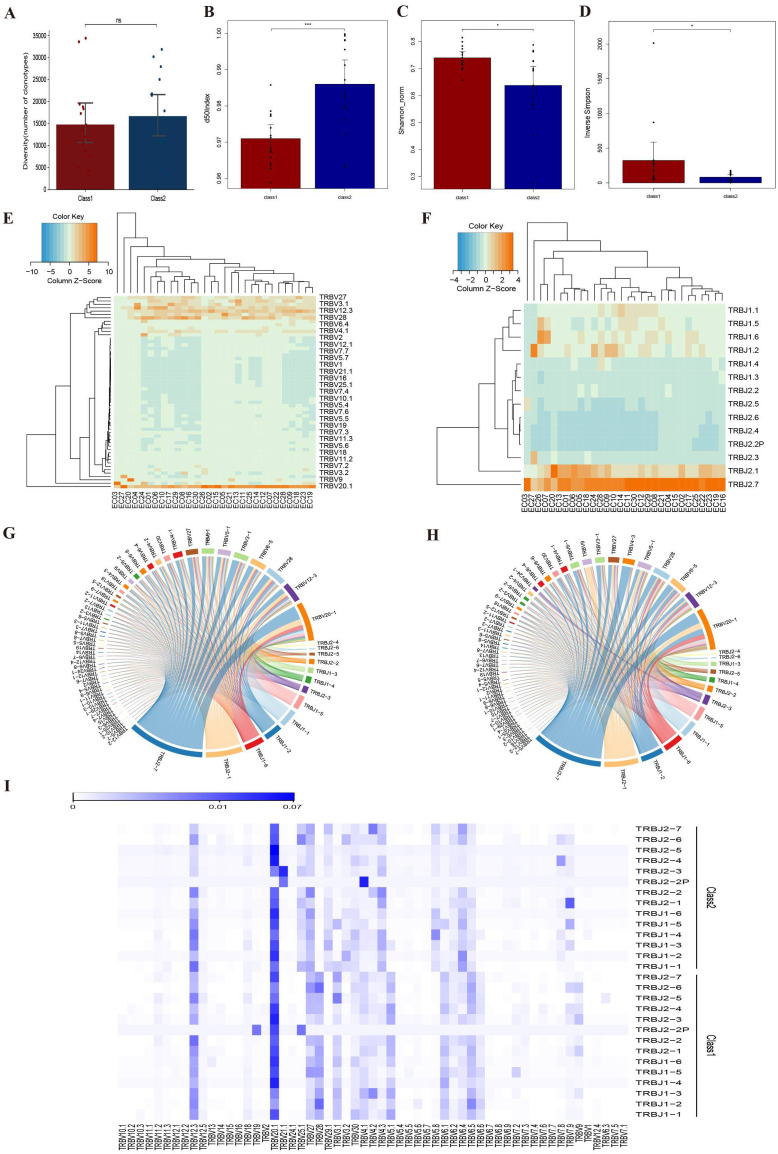
Comparison of TRB diversity between non-ORR group (class 1) and ORR group (class 2). **(A)** Comparison of CDR3 clonotype abundance between the two groups. **(B)** Comparison of d50Index between two groups. **(C)** Comparison of Shannon_norm between two groups. **(D)** Diversity index Inverse Simpson between two groups. **(E)** Frequency of V-gene use in different samples. **(F)** Frequency of J-gene use in different samples. **(G)** V-J linkage circos plot for non-ORR group. **(H)** V-J linkage circos plot for ORR group. **(I)** Heat map of expression of V-J gene linkage frequencies. *:p<0.05, ***:p<0.001, NS, Not Significant.

The abundance of TCR β-CDR3 clones did not differ between and the two groups. We observed that patients in the ORR group had a higher d50 Index and lower Shannon_norm and Inverse Simpson indices than those in the non-ORR group. Moreover, we identified two differential V genes (TRBV29-1 and TRBV4-1) and one differential J gene (TRBJ1-3) in the TRB region. The most frequently connected V genes in both groups were TRBV20-1 and TRBV12-3, while the most frequently connected J genes were TRBJ2-7 and TRBJ2-1. Further analysis revealed differences between the groups: in non-ORR group, the frequency of connected V genes decreased in the following order: TRBV28, TRBV6-5, and TRBV3-1; for J genes, the order was TRBJ1-6, TRBJ1-2, and TRBJ1-1. In ORR group, the frequency for linked V genes decreased in the order of TRBV6-5, TRBV28, and TRBV5-1, while for J genes, the order was TRBJ1-2, TRBJ1-6, and TRBJ1-1 (**Figure 5**).

#### BCR repertoire

Similarly, there were differences in the amino acid composition of CDR3 polypeptide sequences between the ORR and the non-ORR groups within IGH. Substantial oligoclonal enrichment was observed (**Supplementary Figure S3**). The most frequent IGH-CDR3 sequences ranged from 19 to 21 amino acids. Additionally, the sequence pattern of the first three residues was quite similar between the two groups.

Our analysis revealed a significant difference in the clonotype abundance of 9-residue CDR3 polypeptide segments between the two groups. However, no differences were observed in the clonotype abundance of other IGH-CDR3 lengths, nor in diversity comparisons. We identified six differential V genes: IGHV1-45, IGHV3-20, IGHV3-48, IGHV3-49, IGHV4-4, and IGHV5-51, with no significant differences in J genes. The most frequently connected V gene in both groups was IGHV3-21. However, the most frequently connected J gene differed, with TRBJ4 predominating in non-ORR group and TRBJ6 in ORR group. In non-ORR group, the frequency of connected V genes decreased in the order: TRBV3-23, TRBV1-69, and TRBV1-18, while the frequency of connected J genes decreased in the order of TRBJ6, TRBJ3, and TRBJ5. In ORR group, the linkage frequency for V genes decreased in the order of TRBV1-69, TRBV1-18, and TRBV3-23, with linkage J gene frequency decreasing in the order of TRBJ4, TRBJ5, and TRBJ3 (**Supplementary Figure S4**).

## Discussion

In this RWS, we demonstrated that the combination of camrelizumab, an anti-PD1 antibody, with platinum-based chemotherapy is both effective and well-tolerated in the first-line treatment for patients with ESCC. It is noteworthy that the ORR among patients was recorded at 64.8%. Specifically, patients who received camrelizumab plus either the FP or TP chemotherapy regimens exhibited ORRs of 57.9% and 62.3%, respectively. Thus, the percentage of patients who did not show an objective response was 42.1% for the FP regimen and 37.7% for the TP regimen. Although there was no statistical difference between the two regimens, we believe that fluorouracil agents may offer an alternative for patients who are unsuitable for taxanes. However, the ORR obtained from our study aligns with that reported in the KEYNOTE-590 study (pembrolizumab combined with FP, 45%) and the ESCORT-1st study (camrelizumab combined with TP, 72.1%). Accordingly, the percentage of patients who did not exhibit an objective response was 55% for the KEYNOTE-590 study and 27.9% for the ESCORT-1st study. Potential factors influencing the ORR could include treatment regimens, study design, and biomarker analysis. Variations in the specific chemotherapy agents used alongside camrelizumab and dosing schedules may influence the response rates. Additionally, as a RWS, our study may have been subject to diverse patient selection criteria and treatment adherence, in contrast to the controlled settings of RCTs. Furthermore, the lack of comprehensive biomarker analysis, such as PD-L1 expression levels, in our study may contribute to the observed variations in ORRs. Despite the high treatment responses observed across all three studies, we found that approximately 27.9%-55% of patients did not respond to these immunotherapy combinations.

Previous studies have indicated associations between certain biomarkers and the efficacy of ICIs, such as PD-L1 expression (22, 23), tumor mutational burden (24), and microsatellite instability/mismatch repair deficiency (25). However, these markers do not reflect the dynamic changes in immune cell populations during immunotherapy. To explore potential mechanisms underlying differences in treatment responses, we employed IRS in this study. The IR refers to the diversity of T and B lymphocytes within an organism at a given time, representing the immune system’s ability to respond to external stimuli during that period (26, 27). TCRs and BCRs on the surfaces of T and B cells contain CDRs that can specifically recognize and bind to antigenic peptides presented on major histocompatibility complexes, thus activating the immune system. TCRs and BCRs exhibit a high level of diversity generated through somatic recombination and hypermutation, with the CDR3 region serving as the key variable region that determines antigen recognition specificity. The diversity of CDR3 is largely governed by the recombination of V (variable), J (joining), or V, D (diversity), and J gene segments (28, 29). Analyzing the CDR3 regions of TCRs and BCRs might provide a deeper understanding of how specific immune cells function during ESCC treatment and may help identify which genes or sequence variations associated with more effective immune response (30). Studies suggest that the diversity of peripheral blood CD8+ TCRs and dynamic changes in intratumoral TCRs at baseline may serve as effective biomarkers for radiotherapy combined with immunotherapy in ESCC (31).

In immunotherapy, the efficacy of combination treatments is closely linked to changes in T-cell diversity and clonal architecture within the tumor (32). Our study revealed notable distinctions in the TCR and BCR repertoires between patients who responded to camrelizumab plus platinum-based chemotherapy and those who did not. In this study, we found that patients with a treatment response (the ORR group) exhibited a higher d50Index, along with lower Shannon_norm and Inverse Simpson indices. This suggests greater diversity but potentially uneven distribution within the samples, with certain clones being abnormally abundant. These specific T-cell clones might be effectively targeting and eliminating dominant clonal mutations within the tumor cells. This pattern suggests a more robust and targeted immune response in patients who respond to the treatment, with particular T-cell clones playing a key role in identifying and attacking tumor cells. Conversely, in patients without a significant treatment response (the non-ORR group), changes in T-cell diversity may be related to these T-cell clones targeting secondary rather than primary clonal mutations. We identified three differential V/J genes and determined the most frequently linked differential V-J gene pairs, including TRBV29-1, TRBV4-1, and TRBJ1-3, highlighting distinct immune responses between the ORR and non-ORR groups. The unique connectivity patterns observed in the V and J genes underscore the distinct TCR configurations associated with treatment response. Notably, the higher frequency of TRBV6-5 and TRBV28 in the ORR group, along with specific J gene utilization, suggests the presence of TCRs with enhanced capability to recognize and bind to tumor-associated antigens. Our study also highlights the significance of the BCR repertoire in the immune response to camrelizumab combined with platinum-based chemotherapy in ESCC patients. Variations in the amino acid composition and clonotype abundance of IGH-CDR3 segments suggest that certain B-cell clones in the ORR group may be more potent in initiating an immune response against the tumor. The identification of six distinct V genes in the IGH repertoire, particularly in the absence of significant differences in J genes, underscores the vital role of the variable region in determining BCR specificity and affinity for tumor antigens. The distinct connectivity patterns observed in the V and J genes between the ORR and non-ORR groups further emphasize the unique BCR configurations associated with treatment response. Furthermore, the prevalence of TRBJ4 in the non-ORR group and TRBJ6 in the ORR group suggests that specific J genes contribute to the generation of BCRs with varying affinities for tumor-associated antigens. This characterization indicates the potential for specific BCR signatures to serve as predictive biomarkers for treatment response in ESCC. The findings suggest that analyzing the diversity of the TCR repertoire may assist in identifying patients likely to respond to ICI therapy, potentially serving as a predictive biomarker. A favorable treatment response ultimately translates into extended PFS and OS for patients. Future studies will further explore these differences to confirm their predictive value and optimize treatment strategies.

In the ESCORT-1st study, treatment with camrelizumab plus TP included maintenance therapy with a PD-1 antibody for up to two years after six cycles. Although patients in our study did not achieve the ideal duration of treatment, they still experienced survival benefits. Determining the optimal number of cycles for maintenance therapy and rigorously monitoring treatment responses are crucial for early intervention and adapting therapeutic plans for non-responders. It is worth investigating whether TCR repertoire analysis could assist in determining these factors.

Our study has several limitations. Firstly, although we found no differences in efficacy between the TP and FP regimens, particularly regarding treatment response, these results should be interpreted with caution. During the course of our study, the findings from ESCORT-1st study were released and rapidly became the new treatment standard for ESCC. This shift has significantly heightened the preference for the TP regimen among Chinese physicians, resulting in only 19 patients (approximately 20%) receiving the immunotherapy in combination with the FP regimen. Secondly, our analysis of TCR and BCR repertoires was limited to peripheral blood samples due to the unavailability of tumor tissues, and we did not validate our findings with tumor tissues. Thirdly, the use of random selection to match 15 ORR and 15 non-ORR samples, which may introduce bias compared to methods like propensity score matching. Due to the small sample size and limited covariates, PSM was impractical, but future study with larger cohort will address this limitation. Lastly, our study was conducted during the COVID-19 pandemic, which negatively affected the administration of immunotherapy maintenance in patients. Despite these obstacles, nearly 40% of the patients remain alive, suggesting that the optimal duration of immunotherapy maintenance in EC warrant further investigation. Lastly, the differential genes we identified need to be validated in future studies.

## Conclusion

In conclusion, our study demonstrates that camrelizumab combined with platinum-based chemotherapy is an effective and well-tolerated first-line treatment for advanced ESCC. The ORR of 64.8% and 1-year PFS rate of 56.8% highlight the clinical efficacy of this regimen. Furthermore, IRS analysis revealed significant differences in TCR and BCR repertoires between patients with or without ORR. These findings suggest that specific immune repertoire characteristics may serve as predictive biomarkers for treatment response, warranting further validation in larger cohort.

## Data Availability

The data presented in the study are deposited in the Dryad Repository, accession number: https://datadryad.org/stash/dataset/doi:10.5061/dryad.0cfxpnwcf. Related software files are published and publicly available on Zenodo: https://doi.org/10.5281/zenodo.14794837
