# Supplementary material for: Immune repertoire sequencing reveals differences in treatment response to camrelizumab plus platinum-based chemotherapy in advanced ESCC
Source: Front Immunol. 2025 Feb 26;16:1526443. doi: 10.3389/fimmu.2025.1526443 (PMC11897899; doi:10.3389/fimmu.2025.1526443)

**Supplementary Appendix**

**Table of Contents**

Table S1. Grouping information of sequencing samples in the immune repertoire.

Table S2. Analysis of 30 samples differences in clone types between groups for each length in TRB.

Table S3. Statistical table of TRB diversity analysis of 30 samples including diversity, d50Index, Shannon norm, and Inverse Simpson.

Table S4. Analysis of 30 samples differences in clone types between groups for each length in IGH.

Table S5. Statistical table of IGH diversity analysis of 30 samples including diversity, d50Index, Shannon norm, and Inverse Simpson.

Table S6. Analysis of differences in gene expression frequency in TRB and IGH.

**Figures of Contents**

Fig. S1. Treatment effect on overall survival (OS) by subgroup.

Fig. S2. Treatment effect on progression-free survival (PFS) by subgroup.

Fig. S3. Comparison of IGH-CDR3 polypeptide sequences between non-ORR group (class 1) and ORR group (class 2).

Fig. S4. Comparison of IGH diversity between non-ORR group (class 1) and ORR group (class 2).

**Table S1. Grouping information of sequencing samples in the immune repertoire.**

| **Sample ID** | **Class** |
| --- | --- |
| EC01  EC02  EC03  EC04  EC05  EC06  EC07  EC08  EC09  EC10  EC11  EC12  EC13  EC14  EC15  EC16  EC17  EC18  EC19  EC20  EC21  EC22  EC23  EC24  EC25  EC26  EC27  EC28  EC29  EC30 | Class1  Class1  Class2  Class2  Class2  Class1  Class1  Class2  Class2  Class2  Class2  Class2  Class1  Class1  Class1  Class1  Class1  Class2  Class1  Class2  Class2  Class2  Class1  Class2  Class1  Class2  Class2  Class1  Class1  Class1 |

Class1:non-ORR group

Class2:ORR group

**Table S2. Analysis of 30 samples differences in clone types between groups for each length in TRB.**

| **Length** | **P-value** |
| --- | --- |
| 4  5  6  7  8  9  10  11  12  13  14  15  16  17  18  19  20  21  22  23  24  25  26  27  28  29 | 0.39301648  0.065959317  0.540793859  0.177260406  0.349989841  0.308989369  0.547463181  0.566774495  0.633325915  0.547507748  0.623594279  0.594883931  0.566774495  0.682673808  0.533783913  0.520236054  0.566774495  0.771477036  0.506773097  0.31912886  0.146351387  0.28471169  0.63306107  0.466579259  0.314099289  1 |

*p<0.05, statistical difference

**Table.S3 Statistical table of TRB diversity analysis of 30 samples including diversity, d50Index, Shannon norm, and Inverse Simpson.**

| **Sample ID** | **Diversity** | **d50Index** | **Shanno norm** | **Inverse Simpson** |
| --- | --- | --- | --- | --- |
| EC01 | 1223581 | 0.99 | 0.66 | 62.99 |
| EC02 | 2941906 | 0.97 | 0.74 | 98.59 |
| EC03 | 2118372 | 1.00 | 0.28 | 2.62 |
| EC04 | 2534563 | 1.00 | 0.55 | 18.56 |
| EC05 | 2027815 | 0.98 | 0.69 | 51.05 |
| EC06 | 735794 | 0.97 | 0.72 | 93.19 |
| EC07 | 1069464 | 0.98 | 0.70 | 44.46 |
| EC08 | 1334080 | 0.97 | 0.73 | 112.44 |
| EC09 | 1499174 | 0.98 | 0.70 | 171.05 |
| EC10 | 2245037 | 0.96 | 0.77 | 118.30 |
| EC11 | 1989053 | 0.98 | 0.73 | 134.44 |
| EC12 | 3009282 | 0.99 | 0.69 | 149.66 |
| EC13 | 2793043 | 0.98 | 0.70 | 92.03 |
| EC14 | 2100713 | 0.98 | 0.71 | 99.92 |
| EC15 | 2261226 | 0.98 | 0.70 | 50.90 |
| EC16 | 2092677 | 0.96 | 0.77 | 305.48 |
| EC17 | 2532588 | 0.97 | 0.73 | 70.37 |
| EC18 | 1291318 | 0.98 | 0.79 | 169.72 |
| EC19 | 2231050 | 0.97 | 0.75 | 323.73 |
| EC20 | 2830772 | 1.00 | 0.56 | 8.84 |
| EC21 | 2481381 | 1.00 | 0.63 | 49.94 |
| EC22 | 2721196 | 0.96 | 0.76 | 174.55 |
| EC23 | 2244029 | 0.96 | 0.80 | 870.90 |
| EC24 | 1053847 | 0.99 | 0.64 | 35.38 |
| EC25 | 2555609 | 0.96 | 0.81 | 2013.77 |
| EC26 | 2631657 | 1.00 | 0.59 | 20.04 |
| EC27 | 2795822 | 1.00 | 0.45 | 7.47 |
| EC28 | 2887740 | 0.96 | 0.78 | 253.88 |
| EC29 | 1624888 | 0.97 | 0.76 | 278.39 |
| EC30 | 1966913 | 0.97 | 0.76 | 178.65 |

**Table S4. Analysis of 30 samples differences in clone types between groups for each length in IGH.**

| **Length** | **P-value** |
| --- | --- |
| 6  7  8  9  10  11  12  13  14  15  16  17  18  19  20  21  22  23  24  25  26  27  28  29 | 0.305507087  0.073859251  0.073689325  0.018969898*  0.152386502  0.170974004  0.506867669  0.324548175  0.187268401  0.366875192  0.232811909  0.253964081  0.201679436  0.324548175  0.309474306  0.361442952  0.201679436  0.232811909  0.345310897  0.216856639  0.1584183  0.285440871  0.097053886  0.345310897 |

*p<0.05, statistical difference

**Table S5. Statistical table of IGH diversity analysis of 30 samples including Diversity, d50Index, Shannon norm, and Inverse Simpson.**

| **Sample ID** | **Diversity** | **d50Index** | **Shannon Norm** | **Inverse Simpson** |
| --- | --- | --- | --- | --- |
| EC01 | 20472 | 0.97 | 0.76 | 1517.94 |
| EC02 | 15669 | 0.98 | 0.73 | 98.59 |
| EC03 | 5257 | 0.98 | 0.67 | 238.67 |
| EC04 | 22312 | 0.96 | 0.78 | 2025.01 |
| EC05 | 9509 | 0.98 | 0.70 | 474.70 |
| EC06 | 8227 | 0.98 | 0.69 | 386.66 |
| EC07 | 13907 | 0.98 | 0.71 | 650.92 |
| EC08 | 11545 | 0.98 | 0.69 | 519.88 |
| EC09 | 8170 | 0.97 | 0.73 | 553.54 |
| EC10 | 27613 | 0.96 | 0.79 | 2155.44 |
| EC11 | 21457 | 0.97 | 0.75 | 1361.52 |
| EC12 | 27992 | 0.96 | 0.79 | 2545.97 |
| EC13 | 5277 | 0.98 | 0.65 | 207.92 |
| EC14 | 14568 | 0.98 | 0.73 | 904.00 |
| EC15 | 9705 | 0.98 | 0.68 | 411.10 |
| EC16 | 7893 | 0.98 | 0.70 | 443.53 |
| EC17 | 7675 | 0.98 | 0.70 | 408.00 |
| EC18 | 30388 | 0.94 | 0.80 | 2977.80 |
| EC19 | 36642 | 0.95 | 0.80 | 3320.02 |
| EC20 | 51392 | 0.97 | 0.82 | 5517.66 |
| EC21 | 10741 | 0.98 | 0.73 | 638.21 |
| EC22 | 21059 | 0.98 | 0.69 | 42.28 |
| EC23 | 14647 | 0.97 | 0.75 | 1100.15 |
| EC24 | 4646 | 0.98 | 0.66 | 204.71 |
| EC25 | 31634 | 0.97 | 0.77 | 2316.07 |
| EC26 | 14527 | 0.96 | 0.78 | 1349.25 |
| EC27 | 27743 | 0.94 | 0.84 | 3874.67 |
| EC28 | 12100 | 0.98 | 0.71 | 584.71 |
| EC29 | 10326 | 0.98 | 0.71 | 561.26 |
| EC30 | 6258 | 0.98 | 0.68 | 300.82 |

**Table S6. Analysis of differences in gene expression frequency in TRB and IGH.**

| **gene** | **P-value** |
| --- | --- |
| TRBV29.1  TRBV4.1  TRBJ1.3  IGHV1.45  IGHV3.20  IGHV3.48  IGHV3.49  IGHV4.4  IGHV5.51 | 0.001619714*  0.004209946*  0.011400983*  0.013578699*  0.018066623*  0.022531072*  0.012821685*  0.027925057*  0.038088284* |

*p<0.05, statistical difference

**Fig. S1. Treatment effect on overall survival (OS) by subgroup.**


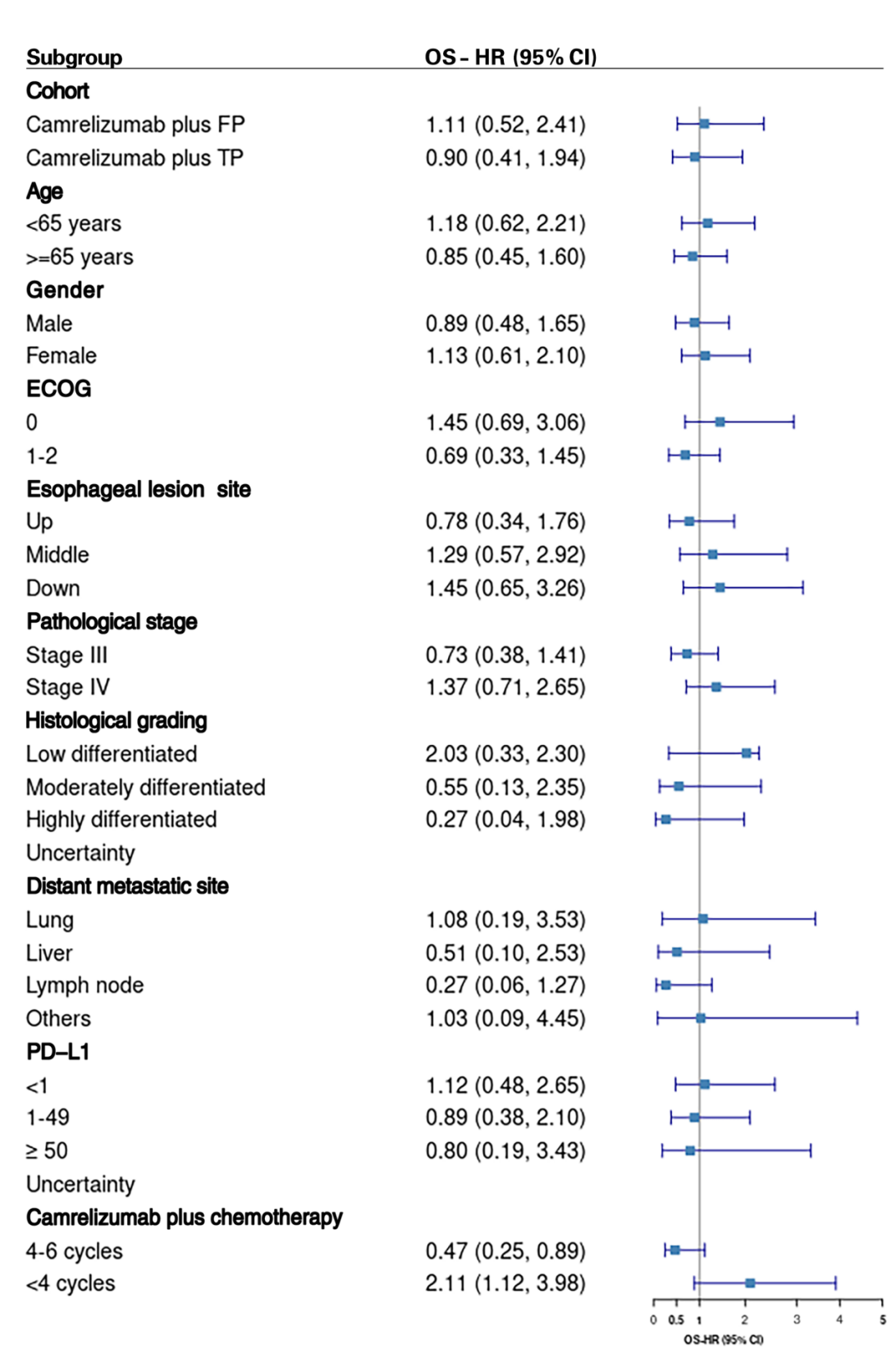


**Fig. S2. Treatment effect on progression-free survival (PFS) by subgroup.**


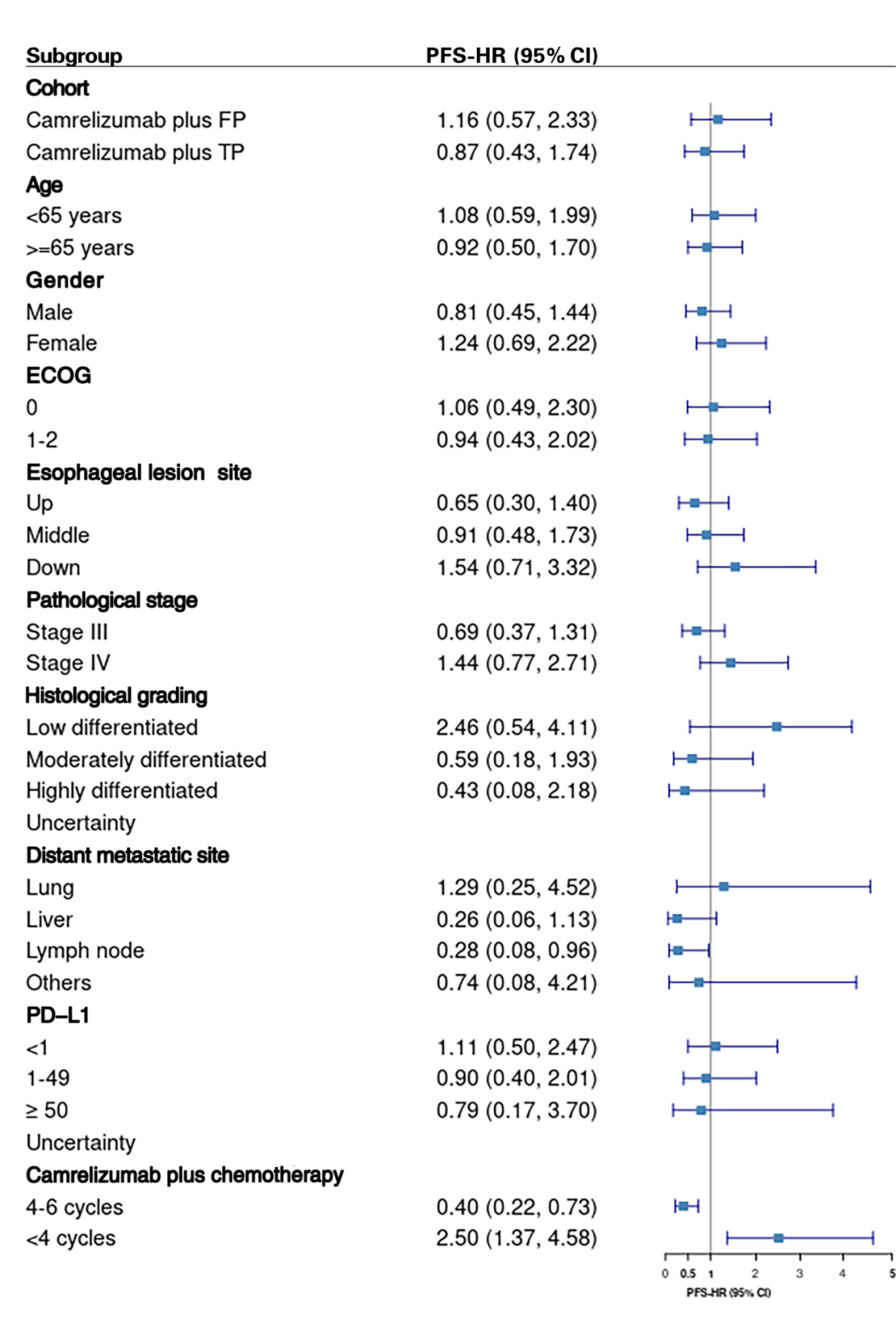


**Fig. S3. Comparison of IGH-CDR3 polypeptide sequences between non-ORR group(class 1) and ORR group(class 2).**

**(A),** Distribution of CDR3 peptide sequence length between groups. (**B)** Comparison of the length distribution of CDR3 peptide sequences between groups. (**C)** Amino acid sequence composition of CDR3 peptides of length 19 in non-ORR group. (**D)** Amino acid sequence composition of CDR3 peptides of length 19 in ORR group. (**E)** Amino acid sequence composition of CDR3 peptide of length 20 in non-ORR group. (**F)** Amino acid sequence composition of CDR3 peptide of length 20 in ORR group. (**G)** Amino acid sequence composition of CDR3 peptides of length 21 in non-ORR group. (**H)** Amino acid sequence composition of CDR3 peptides of length 21 in ORR group.


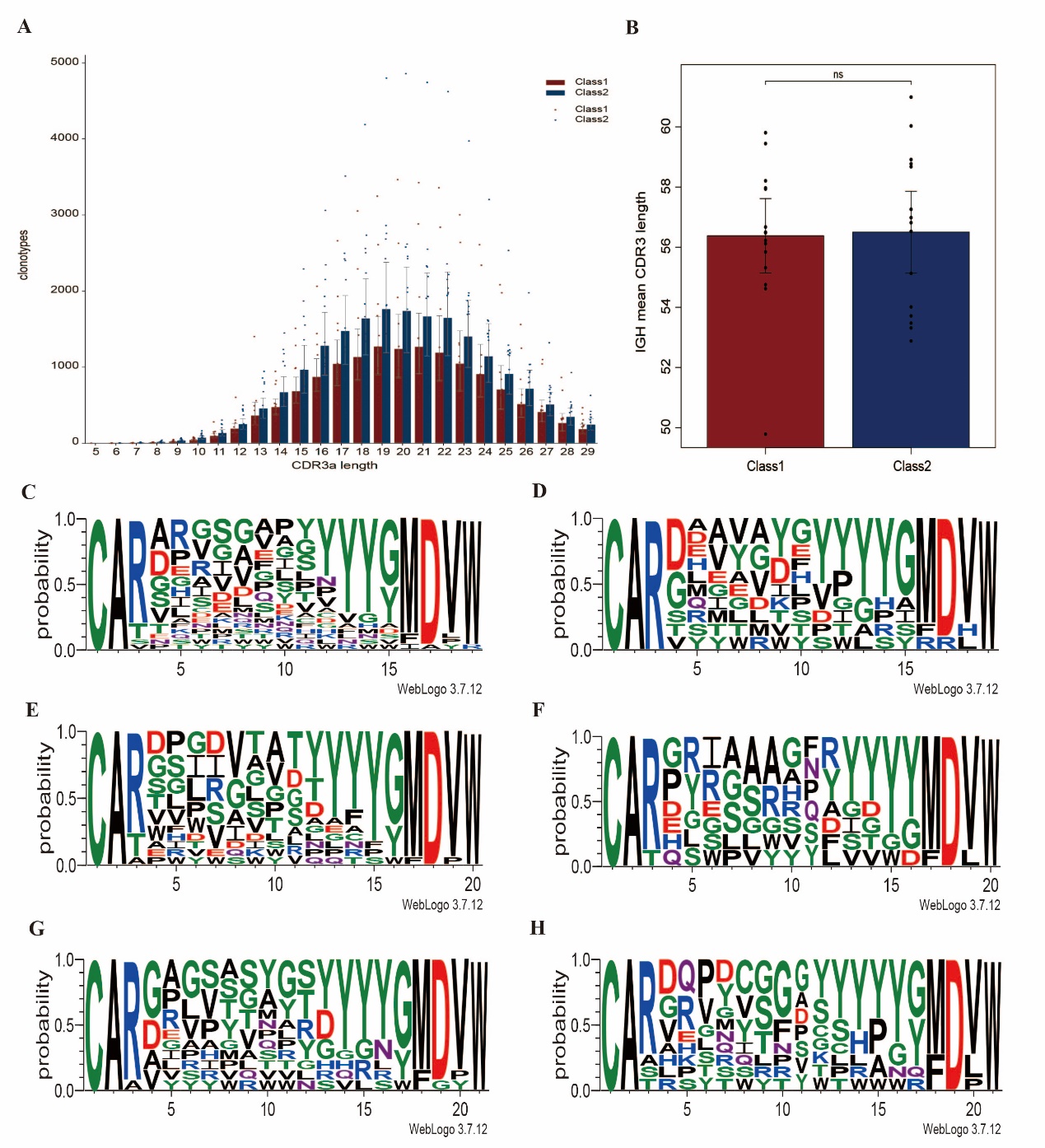


**Fig. S4. Comparison of IGH diversity between non-ORR group(class 1) and ORR group(class 2).**

**(A)** Comparison of CDR3 clonotype abundance between the two groups. (**B)** Comparison of d50Index between two groups. (**C)** Comparison of Shannon_norm between two groups. **(D)**Diversity index Inverse Simpson between two groups. (**E)** Frequency of V-gene use in different samples. (**F)** Frequency of J-gene use in different samples. (**G)** V-J linkage circos plot for non-ORR group. (**H)** V-J linkage circos plot for ORR group. (**I)** Heat map of expression of V-J gene linkage frequencies. (**J)** Sparse curves of IGH diversity for a sample of 30 cases (shaded areas indicate 95% confidence intervals).


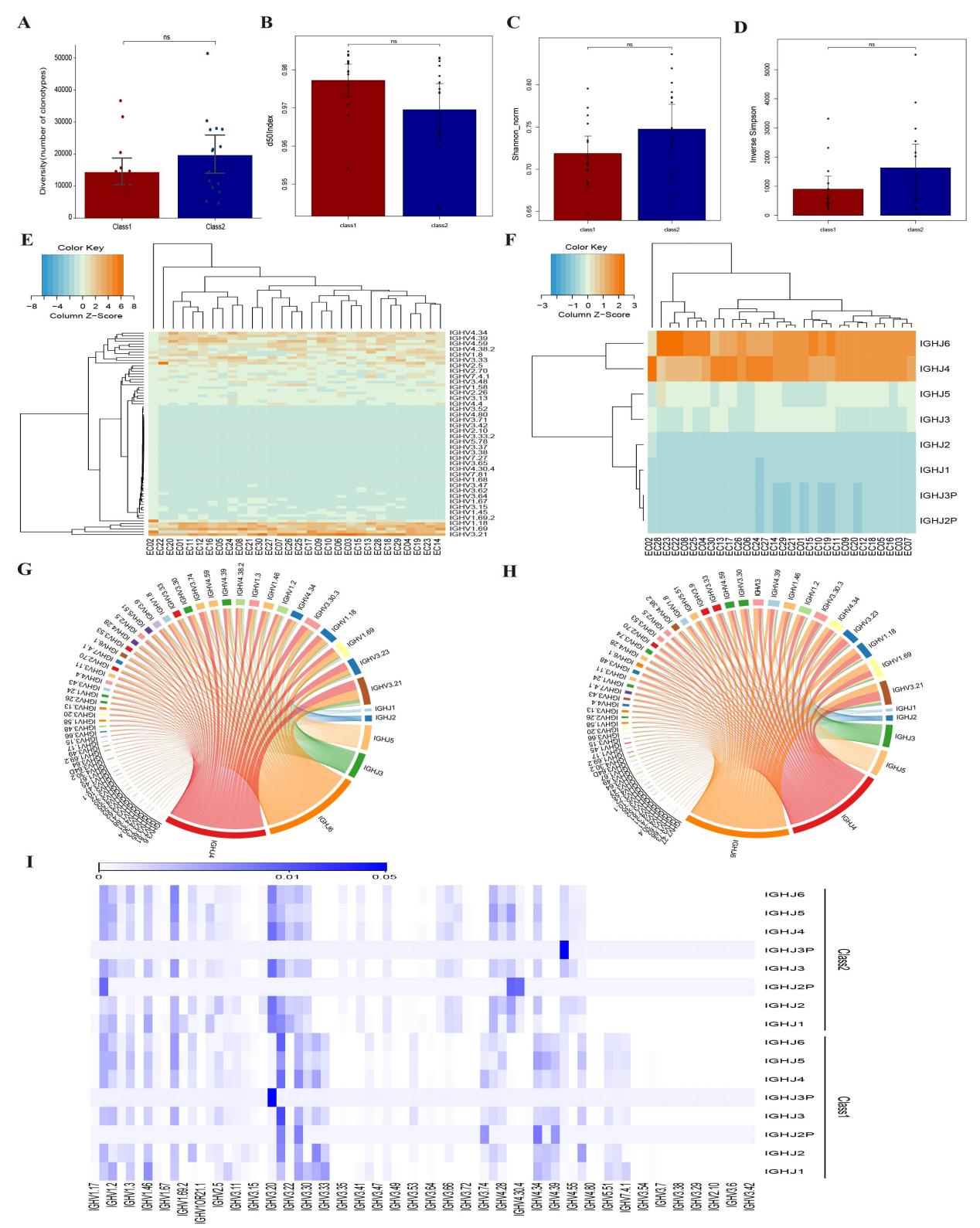

Supplement: Supplementary file 1 [file DataSheet1.docx]
